# Full thickness macular hole formation and spontaneous closure associated with branch retinal vein occlusion in a vitrectomized eye: A case report

**DOI:** 10.1097/MD.0000000000021835

**Published:** 2020-09-04

**Authors:** Ki Yup Nam, Hyun Kyung Cho, Tae Seen Kang, Ji Hye Kim, Bum Jun Kim, Yong Seop Han

**Affiliations:** a Department of Ophthalmology, Gyeonsang National University Changwon Hospital; b Department of Ophthalmology, College of Medicine, Chungnam National University; c Department of Ophthalmology, College of Medicine, Gyeonsang National University, South Korea.

**Keywords:** branch retinal vein occlusion, cystoid macular edema, macular hole, spontaneous closure, vitrectomy

## Abstract

**Rationale:**

Macular hole (MH) formation after vitrectomy is rare and it may be due to several mechanisms associated with change of foveolar anatomy by vitrectomy. If a MH develops after vitrectomy, surgical treatments including internal limiting membrane peeling and intravitreal gas injection are usually needed for repair of hole. Spontaneous closure of MH is much rarer.

**Patient concerns:**

A 66-year-old patient had a vitrectomy for rhegmatogenous retinal detachment not involving the macula of the right eye. Eight months after the vitrectomy, the visual acuity decreased and full-thickness defect of macula, epiretinal membrane progression, intraretinal cysts, and flame shape hemorrhage along with superior temporal vascular arcade were observed on fundus examination and optical coherence tomography.

**Diagnoses:**

MH and branch retinal vein occlusion (BRVO) accompanying cystoid macular edema (CME) were both present on her right eye. Thus, we planned a surgery for MH repair.

**Interventions:**

The status of MH was observed while waiting the surgery schedule. At 2 weeks after detection of the MH, optical coherence tomography showed that intraretinal cysts had decreased in extent and the inner wall of the MH had contracted; 4 weeks later, the MH was closed with a subtle subretinal space.

**Outcomes:**

The fovea was well-maintained with a complete closure for 9 months.

**Lessons:**

MH formation and spontaneous closure occurred in association with BRVO accompanying CME in a patient who had a vitrectomy. In vitrectomized eyes, physicians should consider the possibility of MH development in association with BRVO, and possible spontaneous closure of the MH in accordance with CME resolution.

## 1 Introduction

Macular hole (MH) formation after rhegmatogenous retinal detachment (RRD) repair is not common, with an incidence of approximately 0.5% to 1.9%.^[1–6]^ A MH can occur after scleral buckling, pneumatic retinopexy, and pars plana vitrectomy. Among these procedures, scleral bucking is most closely associated with MH development, with relatively few cases resulting from vitrectomy alone.^[1,3,4]^ In addition, macula-off retinal detachment (RD) and multiple interventions for RD repair have been found to influence MH formation.^[6]^

In most MH cases after vitrectomy, surgical treatments are needed for repair of hole. On the other hand, spontaneous closure of the MH is much rarer. We describe a case of MH formation and spontaneous resolution which occurred associated with branch retinal vein occlusion (BRVO) accompanying cystoid macular edema (CME) after vitrectomy for macula-on RD. The patient has provided informed consent for publication of this case.

## 2 Case presentation

A 66-year-old female patient visited our outpatient clinic due to blurry vision in her right eye. She had hypertension but was free from diabetes mellitus. The best-corrected visual acuity (BCVA) was 1.0 in both eyes, and the intraocular pressure was 19/16. On slit lamp examination, both eyes were pseudophakic, and there were no other specific manifestations in the anterior segment. On fundus examination, the right eye showed ghost vessels in the superior-nasal area around the disc RD, with a large retinal tear at the inferior retina. On spectral domain optical coherence tomography (SD-OCT), localized epiretinal membrane (ERM) not involving the fovea was detected, and the foveal contour was intact. On the same day, a barrier laser was used to treat the RD.

**Figure 1:**
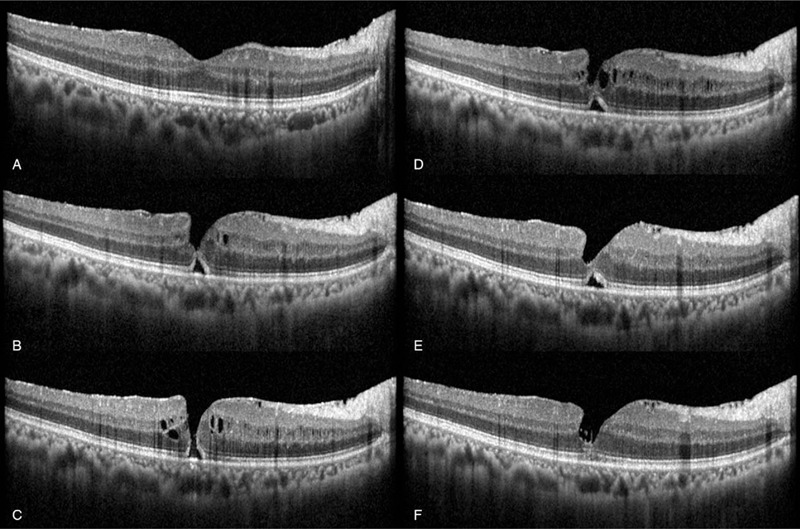
Spectral domain optical coherence tomography of the patient. (A) One month after surgery for rhegmatogenous retinal detachment, ERM which is not involving the fovea was observed (B) Eight months after surgery, the ERM progressed to the macular area and intraretinal cysts and a small MH were noted. (C) At 1 week after initial detection of the MH, the intraretinal cysts had increased in extent and an opened MH was observed. (D) Two weeks after MH detection, the intraretinal cysts had decreased in extent and the inner wall of the hole had contracted. (E) The intraretinal cysts disappeared and the MH was closed with a subtle subretinal space. (F) Nine months after detection, a completely closed MH was noted. ERM = epiretinal membrane, MH = macular hole.

Two months after the initial visit, the RD was found to be enlarged, so surgery including 25-gauge vitrectomy and endolaser gas (18% SF6) injection was performed. One month after surgery, the BCVA of the right eye was well-maintained at 1.0, and the retina was flat. On SD-OCT, the ERM showed no progression (Fig. 1A).

**Figure 2:**
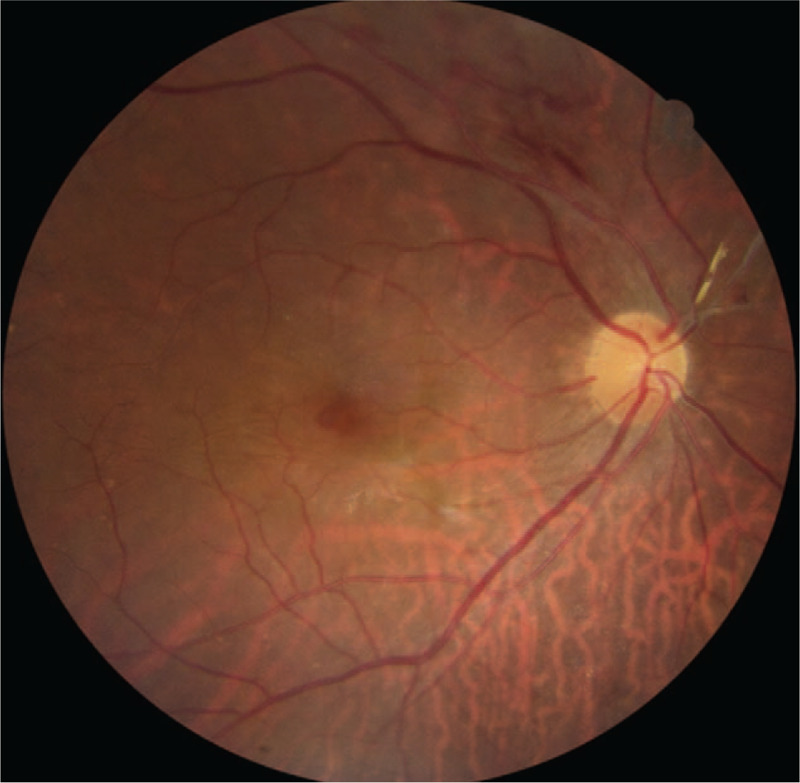
Eight months after a vitrectomy for rhegmatogenous retinal detachment, a flame-shape retinal hemorrhage was observed along the supero-temporal retinal vascular arcade.

However, during the follow-up (several months in duration), the ERM progressed and the visual acuity (VA) worsened. At 8 months after surgery, the BCVA of the right eye was 0.6. On fundus examination, a flame-shape retinal hemorrhage was observed along the superior temporal vascular arcade. (Fig. 2) On SD-OCT, the ERM progressed to the macular area and intraretinal cysts and a small MH were noted (Fig. 1B). We concluded that MH and BRVO were both present. One week later, the MH enlarged, so we planned a surgery (Fig. 1C). However, 2 weeks after detection of the MH, while waiting for surgery, SD-OCT showed that the intraretinal cysts had decreased in extent and the inner wall of the MH had contracted (Fig. 1D). We explained current condition to the patient, and postponed surgery to monitor the status of the eye. Four weeks later, the intraretinal cysts disappeared and the MH was closed with a subtle subretinal space (Fig. 1E).

Nine months after the detection of MH, the VA of the right eye was 1.0. Despite thinning of foveal tissue, the MH was completely closed and did not recur (Fig. 1F). Regarding the BRVO, no associated complications such as macular edema or retinal neovascularization were observed.

## 3 Discussion and conclusion

Idiopathic MH formation is associated with antero-posterior or tangential traction forces on the macular, mediated by the vitreous, and the incidence of MH in eyes with posterior vitreous detachment and vitrectomized eyes is relatively low.^[7]^ Among RRD repair procedures, MH cases developing after scleral buckling have been reported to be the most common; however, MH rarely occurs after vitrectomy alone.^[1,3,4]^ In addition, most cases of MH developing after pars plana vitrectomy for RRD show foveal detachment at the initial diagnosis of RRD,^[6]^ which can be explained by weakening of the fovea due to retinal ischemia and lack of nutrients provided by the retinal pigment epithelium.^[8,9]^

MH formation after vitrectomy has a different pathophysiology to idiopathic MH due to the absence of a tractional force on the macula exerted by the vitreous. Possible mechanisms of MH formation after vitrectomy involve changes in foveolar anatomy, such as ERM formation resulting in a tangential traction force, CME accompanied by coalescence of the perifoveal cystoid spaces, changes in the elasticity of the inner limiting membrane, the traction resulting from intraretinal and preretinal cellular remodeling, and vitreofoveal traction due to residual cortical vitreous.^[7,10–13]^

In the current case, the fovea was attached when RRD was initially diagnosed. Thus, MH formation may be associated with progression of ERM and the occurrence of CME, but not with foveal weakening due to RRD itself. Lee et al^[14]^ reported that CME and ERM are associated with MH formation. They proposed that tangential traction caused by an ERM, and dehiscence of foveal tissue caused by CME may result in a subfoveal cyst, in turn leading to the formation of an MH.

CME after vitrectomy can result from diabetic retinopathy, postoperative inflammation (Irvine-Gass syndrome), and traction of the ERM.^[14,15]^ In our case, there was no history of diabetes mellitus or diabetic retinopathy. Furthermore, while Irvine-Gass syndrome typically onsets within 4 to 6 weeks, CME developed 8 months after vitrectomy for RRD in our case. Although the ERM showed mild progression, it was not sufficiently serious to cause intraretinal cysts. However, when we initially noticed the MH and CME, a flame-shaped hemorrhage along the vascular arcade indicative of BRVO was also noted.

Thus, we assumed that CME due to BRVO caused dehiscence of the foveal tissue, and tangential traction of the ERM opened the gap resulting in a full thickness MH. Because the BRVO was not severe, the CME improved without any specific treatment; the MH closed and the inner wall of the hole contracted.

In conclusion, we report MH formation and spontaneous closure in a patient who had a vitrectomy due to macular-on RRD. We assumed that the course of MH was associated with CME due to BRVO unrelated to the usual post-vitrectomy course. To the best of our knowledge, there has been no similar report. In vitrectomized eyes, physicians should consider the possibility of MH development in association with BRVO, along with possible spontaneous closure of the MH.

## Author contributions

**Conceptualization:** Ki Yup Nam, Yong Seop Han

**Data curation:** Tae Seen Kang, Bum Jun Kim, JI Hye Kim

**Investigation:** Ki Yup Nam, Hyun Jyung Cho, Tae Seen Kang, Yong Seop Han

**Methodology:** Ki yup Nam, JI Hye Kim, Bum Jun Kim, Yong Seop Han

**Project administration:** Hyun Kyung Cho, Tae Seen Kang

**Supervision:** Hyun Kyung Cho, Tae Seen Kang

**Writing – original draft:** Ki Yup Nam, Yong Seop Han

**Writing – review & editing:** Ki Yup Nam, Hyun Kyung Cho, Tae Seen Kang, JI Hye Kim, Yong Seop Han
